# Spinal cord tissue engineering using human primary neural progenitor cells and astrocytes

**DOI:** 10.1002/btm2.10448

**Published:** 2022-11-09

**Authors:** Chen Jin, Yayu Wu, Haipeng Zhang, Bai Xu, Wenbin Liu, Chunnan Ji, Panpan Li, Zhenni Chen, Bing Chen, Jiayin Li, Xianming Wu, Peipei Jiang, Yali Hu, Zhifeng Xiao, Yannan Zhao, Jianwu Dai

**Affiliations:** ^1^ University of the Chinese Academy of Sciences Beijing China; ^2^ State Key Laboratory of Molecular Developmental Biology Institute of Genetics and Developmental Biology, Chinese Academy of Sciences Beijing China; ^3^ Department of Obstetrics and Gynecology The Affiliated Drum Tower Hospital of Nanjing University Medical School Nanjing China

**Keywords:** human spinal cord astrocytes, human spinal cord neural progenitor cells, spinal cord injury, tissue engineering

## Abstract

Neural progenitor cell (NPC) transplantation is a promising approach for repairing spinal cord injury (SCI). However, cell survival, maturation and integration after transplantation are still major challenges. Here, we produced a novel centimeter‐scale human spinal cord neural tissue (hscNT) construct with human spinal cord neural progenitor cells (hscNPCs) and human spinal cord astrocytes (hscAS) on a linearly ordered collagen scaffold (LOCS). The hscAS promoted hscNPC adhesion, survival and neurite outgrowth on the LOCS, to form a linearly ordered spinal cord‐like structure consisting of mature neurons and glia cells. When transplanted into rats with SCI, the hscNT created a favorable microenvironment by inhibiting inflammation and glial scar formation, and promoted neural and vascular regeneration. Notably, the hscNT promoted neural circuit reconstruction and motor functional recovery. Engineered human spinal cord implants containing astrocytes and neurons assembled on axon guidance scaffolds may therefore have potential in the treatment of SCI.

## 1 INTRODUCTION

Spinal cord injury (SCI) is a devastating traumatic injury characterized by the massive loss of neurons and glial cells.^1^ Neural progenitor cell (NPC) transplantation is a promising therapeutic strategy with the potential to replenish lost neurons. Transplantation of NPCs into the injured spinal cord can restore some neural circuits and functions^2^; however, poor retention, survival and integration with host neural circuits limit therapeutic effectiveness.

Functional bioengineered tissues containing multiple cell types are promising SCI therapies. An NPC‐laden scaffold was produced by 3D bio‐printing to promote axon regeneration and function recovery after SCI.^3^ However, engineering spinal cord tissues with longitudinal topological structural features that mimic the parallel axons of the spinal cord remains challenging.

Currently, cells used for spinal cord‐like tissue constructs usually come from rodents.^4^, ^5^, ^6^ Because of differences in cell characteristics and developmental periods between rodent and human spinal cord, therapeutic outcomes in rodents may differ in humans. Human NPCs need a longer time to differentiate into mature neurons than rodent NPCs.^7^, ^8^ Given the short therapeutic window after SCI, accelerating the maturation of human NPCs is pivotal for SCI repair. Gliogenesis occurs after neurogenesis and plays critical roles in the maturation of neurons and the formation of neural circuits.^8^, ^9^, ^10^ Astrocytes, which comprise most of the glial cells in the central nervous system (CNS), are important for maintaining normal activities and for homeostasis.^11^, ^12^ The phenotypes and functions of astrocytes vary with the species, distribution, state of developmental maturity, and environmental triggers.^13^, ^14^, ^15^ Human fetal astrocytes isolated from developing spinal cord may be a good option for establishing a conducive microenvironment for neurons derived from human NPCs (Table 1).

Human spinal‐cord‐like organoids induced from human pluripotent stem cell (hiPSC) have been widely used for investigating human spinal cord development, function, and disease.^16^ However, organoids cannot be directly used for SCI therapy because of their variability, millimeter‐scale sizes, and the tendency to reach a maturational plateau corresponding to mid‐gestational stages.^17^ In addition, because gliogenesis occurs later than neurogenesis,^8^ organoids derived from hiPSCs need long‐term induction to produce mature astrocytes. Until now, the efficiency of acquiring mature astrocytes was low, and constructing human spinal cord organoids containing both neurons and mature astrocytes has been difficult. Without the support of astrocytes, the survival and maturation of neurons and neural circuit reconstruction after transplantation is greatly limited.

In this study, we aimed to optimize the primary culture of human spinal cord cells for neural tissue construction and SCI therapy. We developed methods to culture human fetal spinal cord neural progenitor cells (hscNPCs) and astrocytes (hscAS), and fabricated a centimeter‐scale human neural spinal cord tissue (hscNT) on a linearly ordered collagen scaffold (LOCS). The hscAS promoted hscNPCs survival and axonal outgrowth. The topographical cues in the LOCS induced hscNPCs and hscAS to form a linearly oriented spinal cord‐like structure containing mature neurons and glial cells. When transplanted into rats with complete spinal cord transection, hscNPCs differentiated into mature neurons and formed synaptic connections with host axonal tracts in the hscNT graft, resulting in significant improvement of hindlimb locomotor function in rats with SCI (Table 2).

## 2 RESULTS

### 2.1 Characterization of scaffolds and cells

**FIGURE 1:**
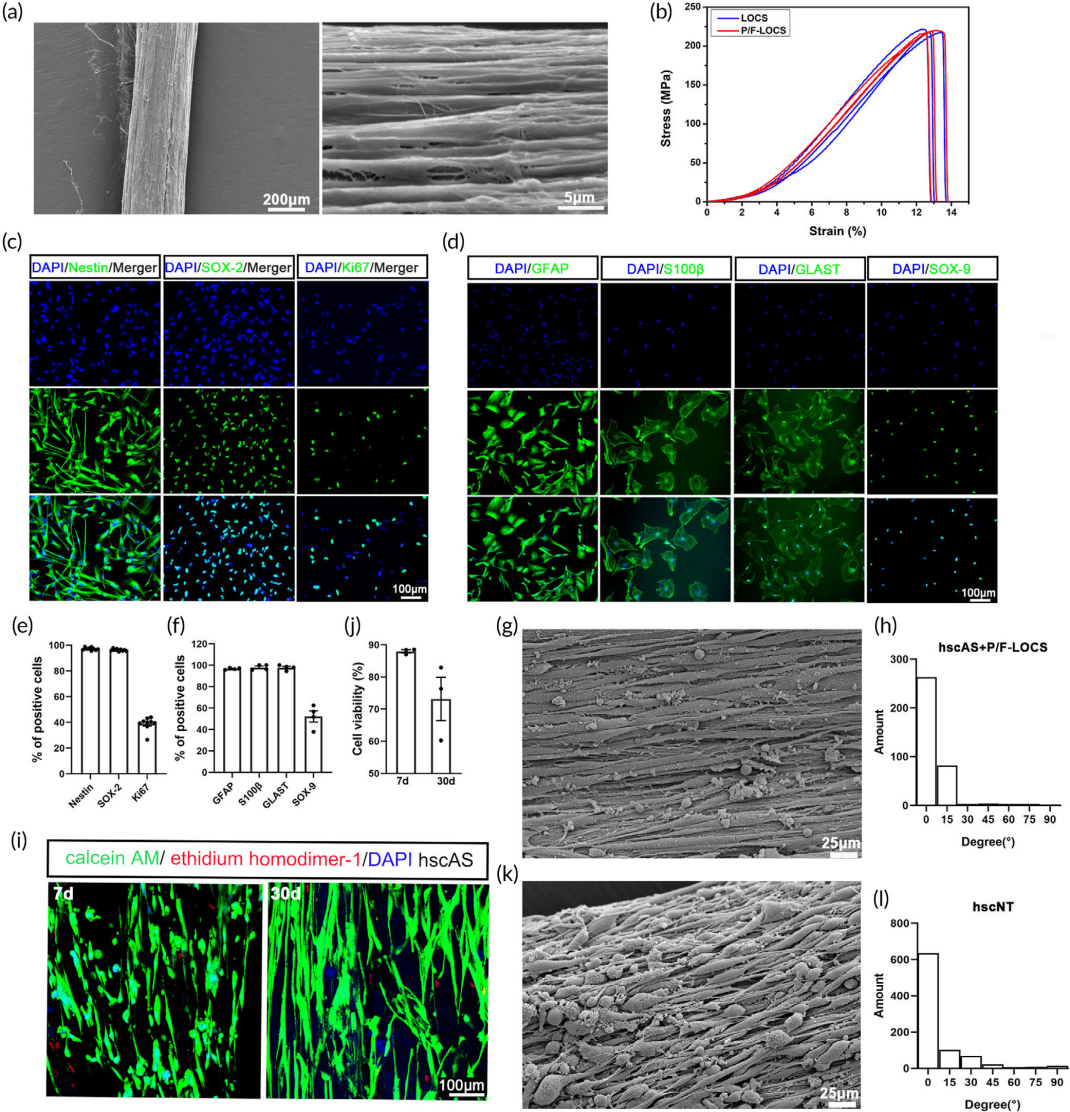
Construction of the hscNT. (a) SEM image showing the linearly ordered structure of the P/F‐LOCS. (b) Stretching stress at various stretching strains for the LOCS and P/F‐LOCS. (c) hscNPCs mainly expressed the stem cell markers Nestin and SOX‐2 and the proliferation marker Ki67. (d) Cultured hscAS expressed astrocyte markers (GFAP, S100β, GLAST and SOX‐9). (e,f) Bar graph showing percentages of cells positive for the different markers (hscNPCs, *n* = 9 images; hscAS, *n* = 4 images). (g) Representative image of hscAS residing on P/F‐LOCS. (h) Statistical analysis of the directionality of hscAS (*n* = 6 images). (i) Viability assays of hscAS on the P/F‐LOCS (green, live; red, dead). (j) Quantification of hscAS viability (*n* = 3 images). (k) SEM image of the hscNT. (l) Statistical analysis of the directionality of total neurites of the cells cultured on the hscNT (*n* = 6 images). Error bars represent standard error.

To achieve greater cell density on the scaffolds, we coated LOCS with 10% poly‐l‐ornithine and 1% fibronectin (P/F‐LOCS). Scanning electron microscopy (SEM) and tension tests showed that the P/F‐LOCS maintained a linearly ordered topological structure (Figure 1a), without changes in mechanical properties (Figure 1b) compared with LOCS. Cell adhesion was greatly enhanced after coating, and 12 h after seeding, hscNPCs on the P/F‐LOCS were almost 9‐fold more numerous than hscNPCs (Figure S1c). By immunofluorescence staining, 97.24 ± 1.1% and 96.33 ± 0.96% of hscNPCs expressed the stem cell markers Nestin and Sox2, respectively, and 39.0 ± 5.11% expressed the proliferation marker Ki67 (Figure 1c,e). The hscNPCs rarely expressed MAP2 (marker for mature neurons), GFAP (marker for astrocytes) or OLIG2 (marker for oligodendrocytes) (Figure S2j,k).

Immunocytochemical analysis revealed that the astrocytes isolated from human fetal spinal cord were highly pure and positive for the astrocyte markers GFAP (96.77 ± 0.87%), S100β (97.99 ± 2.34%), GLAST (97.77 ± 2.36%), and SOX‐9 (52.21 ± 10.49%) (Figure 1d,f). There were almost no DCX^+^ immature neurons, MAP2^+^ mature neurons, Olig2^+^ oligodendrocytes, S100A4^+^ fibroblasts or Iba1^+^microglia among the purified astrocytes (Figure S3a–c). The hscAS grew along the P/F‐LOCS (Figure 1g,h), and more than 85% were viable after 30 days of culture (Figure 1i,j).

The hscNPCs and hscAS exhibited good viability, proliferative capacity, cell size and normal karyology (Figures S2a–e,g and S3d–i) during the passages. Furthermore, the cells did not induce tumors in nude mice, confirming their safety (Figures S2f and S3j). The hscNPCs and hscAS could therefore serve as seed cells for further application.

### 2.2 Construction of the hscNT containing hscNPCs and hscAS

**FIGURE 2:**
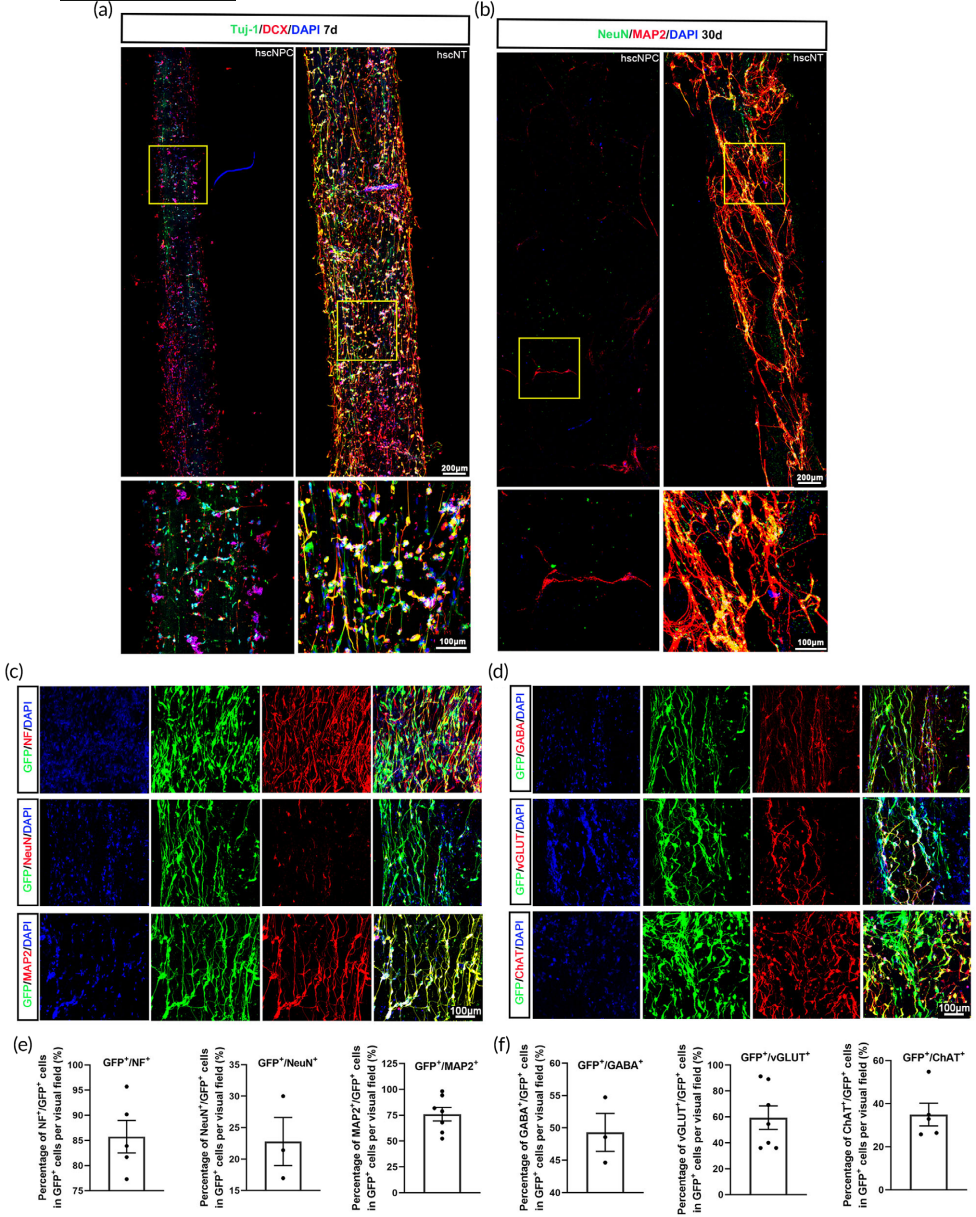
Characterization of the hscNT in vitro. (a) hscNT stained with the neuronal marker Tuj‐1 and DCX. (b) hscNT stained with the mature neuronal marker NeuN and MAP2. (c) hscNPC‐derived cells (GFP^+^) in the hscNT expressing mature neuronal markers (NF, NeuN and MAP2). (d) hscNPC‐derived cells (GFP^+^) in the hscNT expressing GABA (GABAergic marker), vGLUT (excitatory neuronal marker) and ChAT (cholinergic marker). (e) Histogram showing the percentage of NF^+^ (*n* = 5 images), NeuN^+^ (*n* = 3 images) and MAP2^+^ (*n* = 8 images) cells among all GFP^+^ cells. (f) Bar graph showing the percentage of GABA^+^ (*n* = 3 images), vGLUT^+^ (*n* = 8 images) and ChAT^+^ (*n* = 5 images) cells among all GFP^+^ cells. Error bars represent standard error.

Our recent high‐throughput single‐cell analysis of the developing human spinal cord showed that neuronal maturation is accompanied by astrocyte development,^8^ and studies show that astrocytes participate in the development of neurons.^9^, ^18^, ^19^, ^20^ Accordingly, we selected hscNPCs and hscAS to construct an hscNT that mimics the developing spinal cord (Figure S4c). The morphology of hscNPCs and hscAS on the P/F‐LOCS were observed by SEM (Figures 1g,k and S1e). P/F‐LOCS with its oriented structure provided physical guidance for cell growth (Figures 1g,k and S1e). The hscNPCs in the hscNT differentiated into immature neurons (DCX^+^ and Tuj‐1^+^) at 7 days (Figure 2a) and mature neurons (MAP2^+^, NeuN^+^, and NF^+^) at 30 days (Figure 2b,c). ChAT (a cholinergic marker), GABA (a GABAergic marker), and vGLUT (an excitatory neuronal marker) proteins were also detected after 1 month of culture in vitro (Figure 2d). By culturing hscNT in vitro, we found that hscAS was indispensable for hscNPCs adhesion, survival, and outgrowth. hscNPCs alone rarely survived up to 15 days, but they could long‐term survive in the hscNT (Figures 2b and S5a,b). Furthermore, the nerve fibers growing out from the hscNT were longer than those growing out from the hscNPCs alone (Figures 2a,b and S5a,b). The hscAS also promoted hscNPCs adhesion and axon growth in a two‐dimensional culture system (Figure S5c,d). Ca^2+^ is an important intracellular signaling molecule for maintaining normal mammalian nervous system function, and its oscillations can be detected with calcium indicators.^20^, ^21^ Individual cells in the hscNT exhibited spontaneous calcium surges (Movie S1). By contrast, hscNPCs cultured alone did not exhibit Ca^2+^ oscillations (Movie S2).

### 2.3 The hscNT promotes endogenous axon growth after transplantation into rats with SCI

**FIGURE 3:**
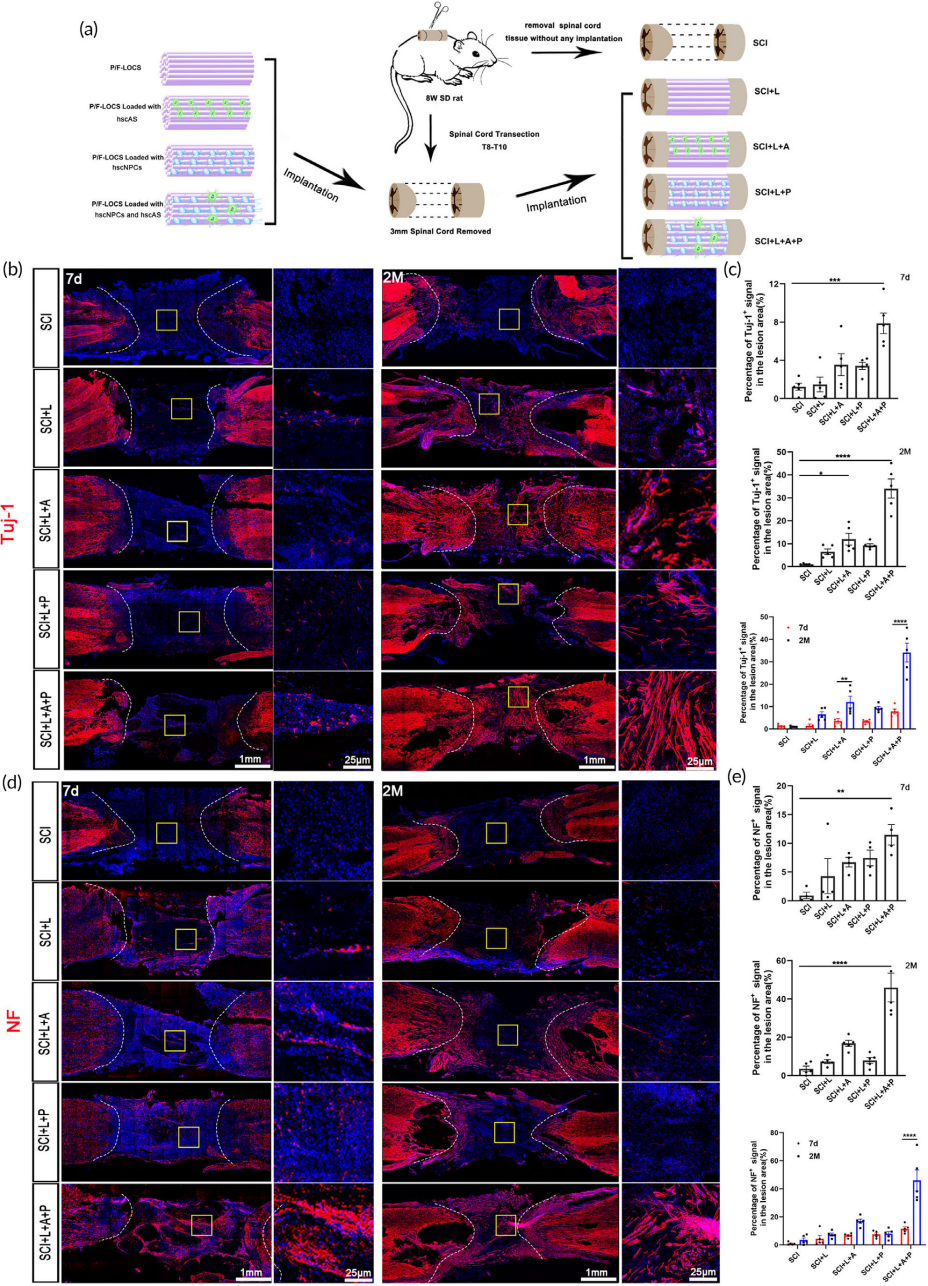
The hscNT promotes axon regeneration after transplantation into rats with complete‐transection SCI. (a) Schematic diagram of transplantation in SCI rats. (b) Tuj‐1 immunostaining in the different groups at 7 days and 2 months post‐injury. (c) Quantification of Tuj‐1^+^ nerve fibers in the lesion core of the spinal cord at 7 days and 2 months (*n* = 5 images). (d) NF immunostaining in the different groups at 7 days and 2 months post‐injury. (e) Quantification of NF^+^ nerve fibers in the lesion core of the spinal cord at 7 days and 2 months (*n* = 4 images). Error bars represent standard error. One‐way analysis of variance, with Tukey's test for post hoc analysis to correct for multiple comparisons. *p < 0.05; **p < 0.01; ***p < 0.001; ****p < 0.0001.

To test the therapeutic effectiveness of the hscNT for SCI, we established a rat complete transection model by removing 3 mm of spinal cord tissue (Figure 3a). First, we investigated the axonal growth of rats in the five groups: SCI (without any implantation, control), SCI + L (P/F‐LOCS graft), SCI + L + A (hscAS loaded on P/F‐LOCS, hscAS graft), SCI + L + P (hscNPCs loaded on P/F‐LOCS, hscNPCs graft) and SCI + L + A + P (hscNPCs and hscAS loaded on P/F‐LOCS, hscNT graft) (Figure 3a). At 7 days, there were few Tuj‐1^+^ and NF^+^ signals in the SCI (1.23 ± 0.367% and 0.93 ± 0.58%), SCI + L (1.27 ± 0.77% and 4.29 ± 3.048%), SCI + L + A (3.55 ± 1.15% and 6.72 ± 0.83%), SCI + L + P (3.43 ± 0.38% and 7.46 ± 1.37%) and SCI + L + A + P (7.87 ± 1.07% and 11.50 ± 1.78%) groups (Figure 3b–d). The density of axons within the lesion site increased over time in the five groups, and there were significant differences between the SCI + L + A (Tuj‐1^+^, 12.02 ± 2.57%, *p* < 0.01) and SCI + L + A + P (Tuj‐1^+^, 34.08 ± 4.19%, *p* < 0.0001 and NF^+^, 46.00 ± 7.47%, *p* < 0.0001) groups (Figure 3b–d). These results indicate that hscAS promote neurogenesis or axon growth.

### 2.4 The hscNT creates a favorable biological microenvironment for SCI repair

**FIGURE 4:**
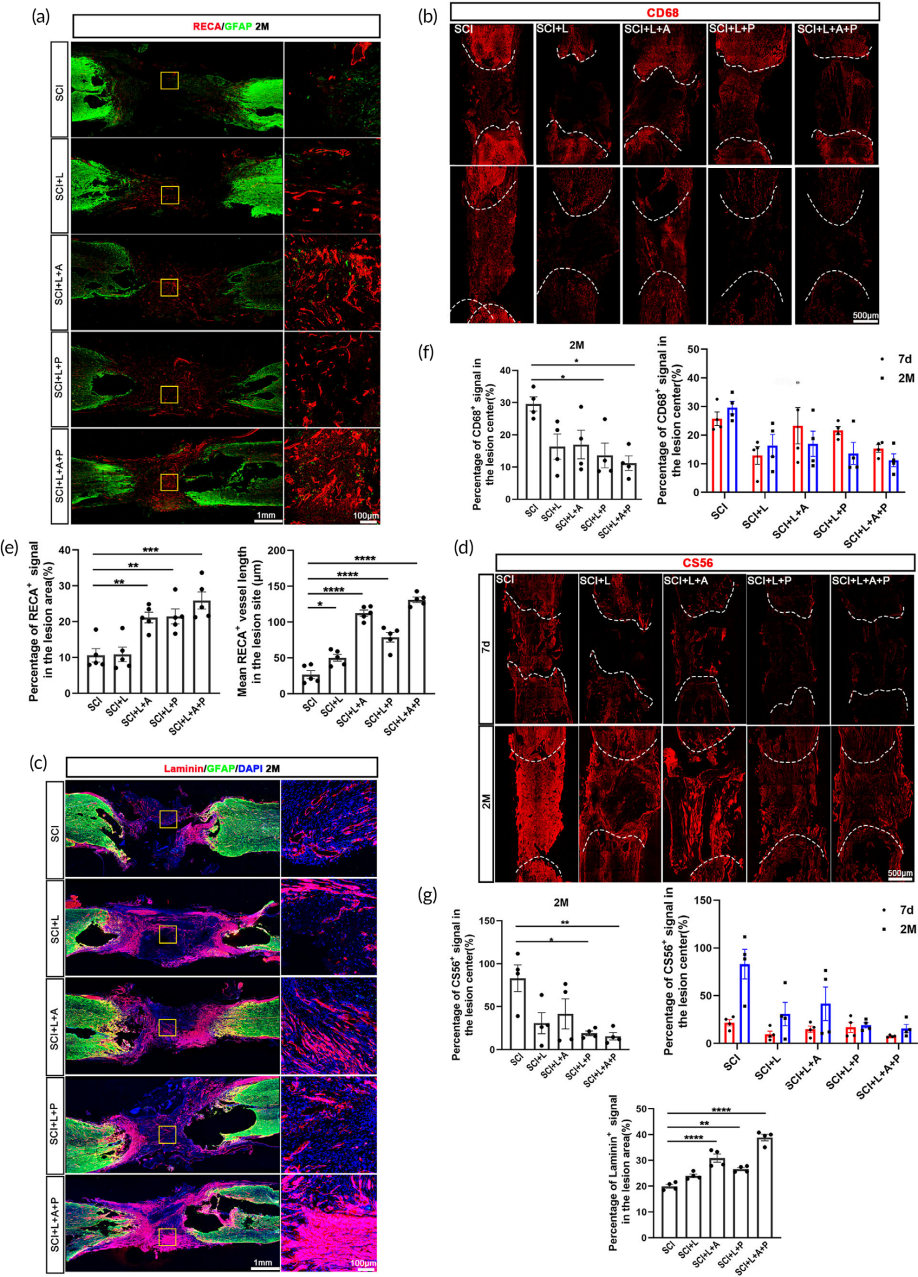
The hscNT creates a favorable microenvironment for neural regeneration at the injury site. (a) Regeneration of RECA^+^ blood vessels at the lesion site in SCI rats. (b) Immunofluorescence staining for CD68 shows inflammatory cell infiltration. (c) Deposition of laminin at the lesion site in SCI rats. (d) Images of CS56 immunostaining show deposition of CSPGs at the lesion sites. (e–g) Quantification of blood vessel regeneration (e) (*n* = 5 images), activated microglia/macrophage infiltration (f) (*n* = 4 images), and laminin and CS56 protein deposition at the lesion site in the different groups (g) (*n* = 4 images). Error bars represent standard error. One‐way analysis of variance was performed, and Tukey's test was used for post hoc analysis to correct for multiple comparisons. *p < 0.05; **p < 0.01; ***p < 0.001; ****p < 0.0001.

Promoting vascular regeneration within the lesion site is beneficial for axon regeneration and SCI repair.^22^ We therefore analyzed the revascularization profiles in each group. Rats in the SCI + L + A + P group had the most blood vessels (25.92 ± 2.39%) in the lesion site, which were identified by immunostaining with RECA (Figure 4a,e). The blood vessels in the SCI + L + A + P group were longer (131.20 ± 3.73 μm) than those in the other groups (Figure 4a,e). The SCI + L + A and SCI + L + P groups had the second highest number of blood vessels (21.16 ± 1.49% and 21.49 ± 2.07%), and their blood vessels were substantially longer (112.6 ± 4.27 μm and 78.80 ± 6.74 μm) than those in the SCI (26.8 ± 5.48 μm) group (Figure 4a,e). These results indicate that the hscNT had a better effect than hscAS or hscNPCs on vascular regeneration after SCI.

Next, we investigated the infiltration of inflammatory cells after injury by immunostaining for CD68. CD68 expression in the SCI + L + A + P, SCI + L + P and SCI + L + A groups decreased over time. In the SCI + L + A + P and SCI + L + P groups, CD68^+^ signals (11.23 ± 2.25% and 13.61 ± 3.83%) were lower than those in the SCI group (29.59 ± 2.21%) at 2 months, indicating that transplantation of hscNPCs and the hscNT effectively reduces the inflammatory response at the injury site (Figure 4b,f). Moreover, rats in the SCI + L and SCI + L + A groups also had relatively lower expression levels of CD68 (16.34 ± 3.92% and 16.99 ± 4.42%) than those in the SCI group at 2 months, indicating that P/F‐LOCS and hscAS also play important roles in reducing inflammatory cell infiltration (Figure 4b,f).

Finally, we examined two major extracellular matrix (ECM) proteins at the lesion site—laminin (LN), which enhances neuronal survival and promotes neurite growth,^23^ and chondroitin sulfate proteoglycan (CSPG), which inhibits neurite growth and facilitates glial scar formation. LN expression was the highest in the transplanted region in the SCI + L + A + P group (38.87 ± 1.28%), followed by the SCI + L + A (30.89 ± 1.60%) and SCI + L + P (26.64 ± 0.58%) groups, but was only weakly expressed in the SCI group (19.98 ± 0.73%) (Figure 4c,g). The areas positive for CS56 (a marker for CSPG) in the SCI + L + A + P (15.58 ± 4.25%), SCI + L + P (19.07 ± 2.70%), SCI + L (30.78 ± 12.23%) and SCI + L + A (41.60 ± 17.53%) groups were reduced compared with the SCI group (83.09 ± 15.53%) at 2 months. The deposition of CSPG in the SCI + L + A + P group was the lowest among the five groups, both at 7 days and 2 months (Figure 4d,g). These results suggest that hscNT transplantation creates a more favorable biological microenvironment by regulating ECM composition at the injury site, which might be beneficial for neuronal survival and neurite growth.

### 2.5 The hscNPCs differentiate into mature neurons in vivo

**FIGURE 5:**
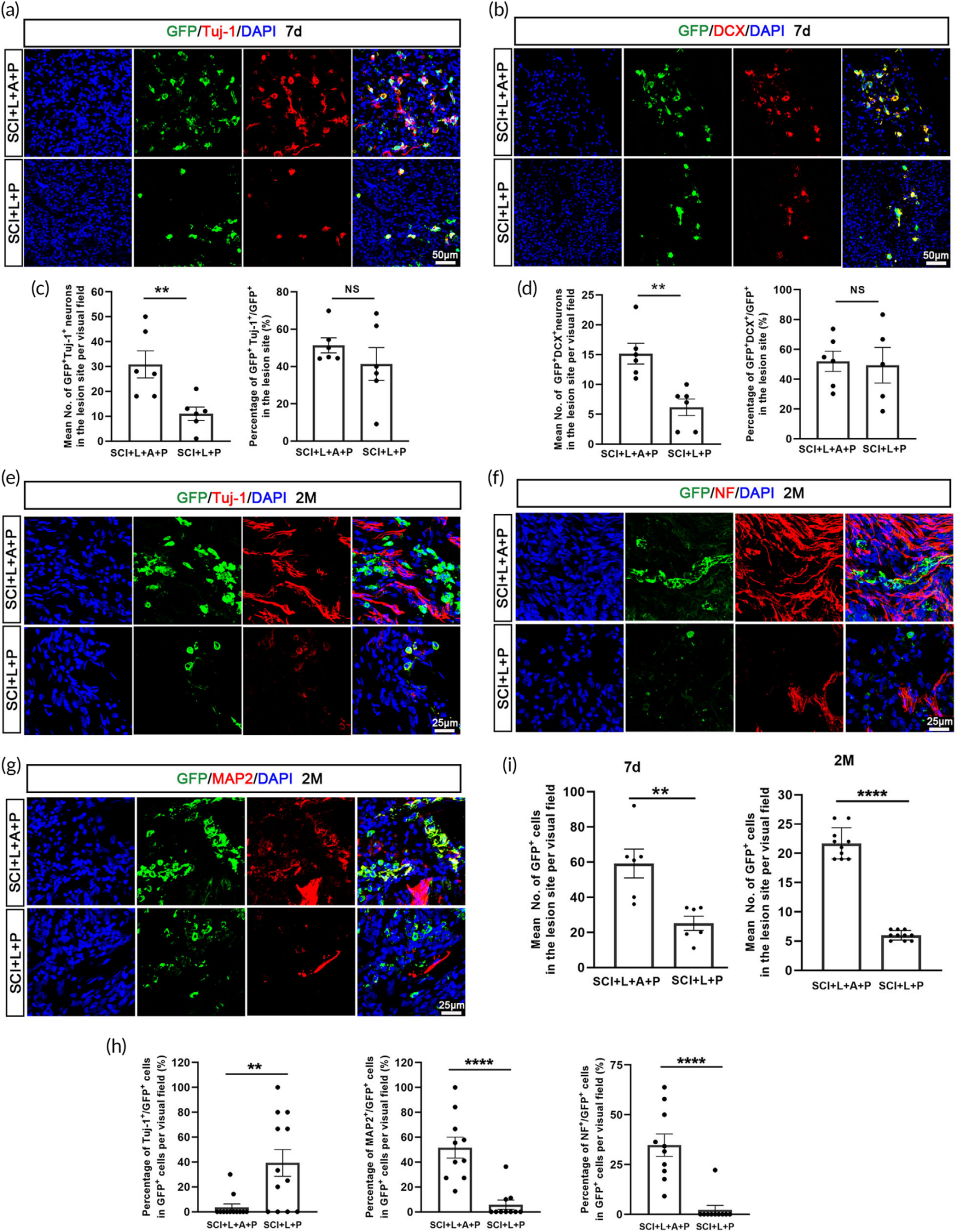
The hscNT promotes cell survival and neuronal maturation in the lesion center. (A and B) hscNPCs differentiate into neurons expressing the immature neuron markers Tuj‐1 (a) and DCX (b) at 7 days. (c) Bar chart showing the percentage of Tuj‐1^+^/GFP^+^ and DCX^+^/GFP^+^ cells among all GFP^+^ cells in the two groups at 7 days. (d) Bar chart showing the numbers of Tuj‐1^+^/GFP^+^ and DCX^+^/GFP^+^ cells in the two groups at 7 days (*n* = 6 images). (e,g) Immunofluorescence staining images showing Tuj‐1^+^ immature neurons (e) and NF^+^ (f) and MAP2^+^ (g) mature neurons in the injury center at 2 months. (h) Quantification of the percentage of Tuj‐1^+^/GFP^+^, NF^+^/GFP^+^ and MAP2^+^/GFP^+^ cells among all GFP^+^ cells in the SCI + L + P and SCI + L + P + A groups at 2 months (*n* = 10 images). (i) Quantification of the numbers of grafted GFP^+^ cells in the lesion center at 7 days (*n* = 6 images) and 2 months (*n* = 10 images). Error bars represent standard error, and two‐group comparisons were analyzed using Student's *t*‐test. NS indicates no significant difference. *p < 0.05; **p < 0.01; ***p < 0.001; ****p < 0.0001.

We next quantified the numbers of GFP^+^ cells per visual field. In the SCI + L + A + P group, at 7 days and 2 months, GFP^+^ cells (59.17 ± 8.20% and 21.70 ± 0.84%, respectively) were significantly more numerous than in the SCI + L + P group (25.17 ± 4.01% and 6.00 ± 0.26%, respectively) (Figure 5i).

Next, we examined the differentiation of hscNPCs after transplantation. At 7 days, some GFP^+^ cells differentiated into neurons expressing Tuj‐1 (Figure 5a) and DCX (Figure 5b). The percentages of Tuj‐1^+^/GFP^+^ cells were similar in the SCI + L + P (79.97 ± 4.91%) and SCI + L + A + P (84.80 ± 1.73%) groups (Figure 5c). However, a higher proportion of DCX^+^GFP^+^/GFP^+^ cells were detected in the SCI + L + A + P group (82.05 ± 2.31%) compared with the SCI + L + P group (71.75 ± 2.33%) (Figure 5c). Additionally, the numbers of Tuj‐1^+^/GFP^+^ and DCX^+^/GFP^+^ donor cells in the SCI + L + A + P group (50.67 ± 7.59% and 26.33 ± 4.79%, respectively) were significantly greater than those in the SCI + L + P group (18.33 ± 3.96% and 11.67 ± 3.59%, respectively) (Figure 5d). These results suggest that more hscNPC‐derived neurons were localized at the injury site in the SCI + L + A + P group than in the SCI + L + P group. At 2 months, rats in the SCI + L + P group had a relatively higher proportion of Tuj‐1^+^/GFP^+^ cells (39.31 ± 10.76%) at the injury site compared with the SCI + L + A + P group (3.69 ± 2.67%); however, the proportions of NF^+^/GFP^+^ and MAP2^+^/GFP^+^ cells were both substantially higher in the SCI + L + A + P group (34.74 ± 5.62% and 51.67 ± 8.40%, respectively) than in the SCI + L + P group (2.22 ± 2.22% and 5.75 ± 3.67%, respectively) (Figure 5e–h), suggesting that hscNPC‐derived neurons were more mature in the SCI + L + A + P group. These findings indicate that, compared with hscNPCs transplantation, hscNT transplantation provides a larger number of mature neurons.

### 2.6 The hscNT forms synaptic connections with host cells

**FIGURE 6:**
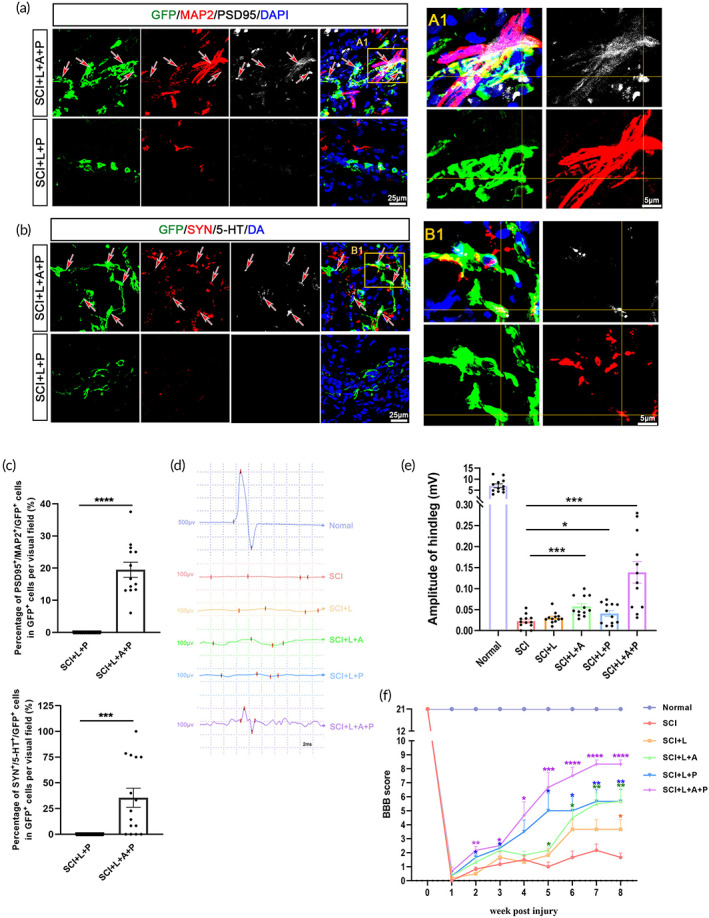
The hscNT structurally integrates with the host neural circuits and effectively improves hindlimb motor function. (a) Host dendrites (GFP^−^/MAP2^+^) regenerate into the lesion site and form appositional contacts (arrows) and synapses (PSD95) with the implanted donor neurons (GFP^+^). (A1) Enlarged image of synaptic junctions between host dendrites and the hscNT. The areas in the yellow boxes show host dendrites. (b) 5‐HT‐labeled host serotonergic axons enter the lesion center and establish a synaptic junction (SYN) with the donor neuron (labeled with GFP). (B1) Enlarged image showing 5‐HT^+^ axons regenerated in the lesion site and contacting the hscNT. (c) Quantification of synaptic connections between host cells and transplanted cells (GFP^+^/MAP2^+^/PSD95^+^, *n* = 13 images; GFP^+^/5‐TH^+^/SYN^+^, *n* = 15 images). (d) CMEP responses in the different groups. (e) Histogram showing that rats implanted with the hscNT have greater CMEP amplitudes (*n* = 12 images). (f) BBB motor scores after hscNT transplantation in rats with SCI (*n* = 6 rats). Error bars represent the standard error. Two‐group comparisons were analyzed using Student's *t*‐test, and multiple groups comparisons were analyzed using one‐way analysis of variance with Tukey's test. NS indicates no significant difference, *p < 0.05; ***p < 0.001; ****p < 0.0001.

In our experiment, numerous GFP^+^ neurons formed contacts with host MAP2^+^/GFP^−^ neurons in the SCI + L + A + P group, and some of these cells were PSD95^+^ (19.50 ± 2.33%), suggesting synaptic connections between the hscNT and host neurons (Figure 6a,c). However, there were no MAP2^+^/PSD95^+^/GFP^+^ neurons in the SCI + L + P group (Figure 6a,c).

5‐HT nerve fibers descending from the brainstem are important for lower limb movements. In the SCI + L + A + P group, we observed 5‐HT^+^ nerve fibers at the injury site, and some synapses formed between these fibers and the GFP^+^ donor cells (35.50 ± 9.26%) (Figure 6b,c). In the SCI + L + P group, 5‐HT^+^ nerve fibers rarely extended into the injury center (Figure 6b,c).

### 2.7 Transplantation of the hscNT effectively enhances hindlimb motor functional recovery

Cortical motor‐evoked potentials (CMEPs) are widely used for SCI evaluation. The hscNT grafts increased the amplitudes of CMEPs at 8 weeks in the SCI + L + A + P group (0.139 ± 0.026 mV) compared with the other groups (Figure 6d,e). Rats in the SCI + L + P (0.040 ± 0.007 mV) and SCI + L + A (0.057 ± 0.007 mV) groups both recovered better than those in the SCI (0.022 ± 0.004 mV) and SCI + L groups (0.030 ± 0.004 mV) (Figure 6d,e). The Basso, Beattie, and Bresnahan (BBB) open‐field locomotor test was used to assess hindlimb motor function. The test revealed better motor function in the SCI + L + A + P and SCI + L + P groups compared with the other three groups from 5 weeks after transplantation (Figure 6f). The BBB score for rats in the SCI + L + A + P group was greater than 9 points at 7 weeks, indicating weight‐bearing locomotion (Figure 6f). Overall, the rats in the SCI + L + A + P group showed significant improvement in hindlimb motor function and electrophysiological features compared with the other four groups.

## 3 DISCUSSION

With significant advances in tissue engineering, neural tissue construction is emerging as a promising approach for SCI repair.^3^, ^4^, ^5^, ^24^ In this study, we constructed a novel spinal cord‐like neural tissue with human fetal spinal cord‐derived NPCs and astrocytes and demonstrated its therapeutic effectiveness in the rat complete SCI model. The hscNT allowed hscNPCs–hscAS–ECM interactions and recapitulated the developmental spinal cord microenvironment. The hscNPCs differentiated into specific neuronal subtypes. After transplantation in vivo, the hscNT formed synaptic connections with the host tissue and created a favorable biological microenvironment for neural regeneration, thereby dramatically improving hindlimb motor function.

Unlike transplantation of freshly isolated stem cells,^25^, ^26^ we constructed a transplantable bioengineered human neural spinal cord tissue to replace lost spinal cord tissue. The microenvironment formed by the hscNT during 3D coculturing conferred greater cellular resilience in the harsh SCI milieu. In addition, the linearly oriented biomaterial scaffolds efficiently promoted axonal growth and synapse formation. Recently, neural organoids generated from hiPSCs have shown the potential to mimic cell‐type composition and cytoarchitecture, offering new prospects in biomedical research and neurodevelopmental biology.^16^, ^27^ Although 3D nerve organoids can imitate some features of the spinal cord unit by spinal‐cell‐type induction, dorsoventral specification and 3D trunk neuromuscular connections,^28^, ^29^, ^30^ batchwise and intra‐ or interorganoid variations, millimeter‐scale sizes, simple patterns and internal dense avascular structures have limited their use in transplantation for spinal cord injury.^31^ To the best of our knowledge, the present study is the first to report the transplantation of organoids for the treatment of spinal cord injury. The hscNT is the first engineered human spinal cord implant containing astrocytes and neurons assembled on axon guidance scaffolds that show great potential for SCI treatment.

Recent SCI cell therapy studies show that grafting with spinal cord‐derived cells is crucial for spinal cord “replacement.” Dell'Anno et al. showed that human neuroepithelial stem cells (NES) derived from the developing spinal cord successfully form a relay system through the lesioned area reconnecting separated host neural elements.^32^ Similarly, Kadoya et al. discovered that driving neural stem cells toward caudalized, rather than rostralized, fates, or isolating NPCs from developing spinal cords results in robust regeneration of corticospinal axons and the formation of functional excitatory synapses.^33^ Furthermore, in our recent work, human NPCs derived from fetal spinal cord displayed better therapeutic outcome than those from brain.^34^ Collectively, these observations reveal that anatomical matching of the graft with recipient tissue is a key factor in functional neuronal network formation.

Astrocytes provide physical support for neuronal migration and axonal pathfinding,^35^, ^36^, ^37^, ^38^ support neuritogenesis and synaptogenesis,^39^, ^40^, ^41^ and regulate metabolic and trophic activity.^42^ These properties underlie the rationale for using astrocytes to construct neural tissue for SCI repair. Nonetheless, human astrocyte culture is time consuming and technically challenging.^11^, ^43^, ^44^, ^45^ Zhang et al. successfully used immunopanning to acquire human spinal cord astrocytes for transcriptional and functional studies.^46^ Here, we described a new protocol without immunopanning to efficiently isolate astrocytes from spinal cord. hscAS with a purity of up to 95% were obtained, and they could be passaged long‐term for tissue construction and transplantation therapy.

LOCS have been used in clinical studies, and their safety and feasibility have been demonstrated.^47^, ^48^ In the present study, we modified LOCS with poly‐l‐ornithine and fibronectin, which increased the number of hscNPCs residing in the scaffold without changing the linearly ordered structure or mechanical properties. The neurites of the hscNPCs in the P/F‐LOCS extended directly along the aligned scaffolds, just as in the normal spinal cord. The physical cues in the P/F‐LOCS impacted hscAS morphology and directed the formation of an aligned astrocytic network. Aligned astrocytic networks emulate the neuroanatomy and physiology of astrocytes in vivo, which could facilitate neurite extension into the injured spinal cord. As shown in this study, regeneration of Tuj‐1^+^ and NF^+^ fibers in the hscAS transplantation groups (SCI + L + A and SCI + L + A + P groups) was significantly enhanced compared with the other groups.

The crosstalk between neurons and astrocytes contributes to neural tissue development and information processing.^10^, ^18^ In this study, although hscNPCs could differentiate into neurons when grafted alone, the newborn neurons at the site exhibited several features of immature neurons. After the hscAS and hscNPCs were seeded together on the LOCS, the former supported the survival of the latter and accelerated their maturation. More NF^+^ and MAP2^+^ neurons were observed at the injury site, and some neurons formed synapses with host neural cells. Recent studies shown that neurons and neuron‐derived neuroactive compounds influence the development and function of astrocytes by regulating their gene expression.^49^, ^50^ Therefore, we propose that the interaction between hscNPCs and hscAS might be critical for SCI repair.

Here, locomotor and neuroelectrophysiological functions were improved after hscNT implantation, suggesting that preconstructing a spinal cord‐like tissue with a linear structure may be a promising strategy to enhance therapeutic outcome for SCI. The regeneration of neurites of neurons in the hscNT were limited in vivo compared with in vitro culture. Compared with the in vitro microenvironment, the site of SCI in vivo contains large amounts of myelin‐associated proteins, which have been reported to inhibit axon extension by activating endogenous signaling pathways.^51^, ^52^ Inflammation and reactive oxygen species might also contribute to the adverse microenvironment at the injury site. Therefore, further improving the microenvironment for hscNT transplantation at the lesion core should be an objective of future studies.

## 4 MATERIALS AND METHODS

### 4.1 Preparation and characterization of P/F‐LOCS

The LOCS was prepared as previously described.^53^ 10% poly‐l‐ornithine (P4957, Millipore) and 1% fibronectin (F0895, Sigma‐Aldrich) were coated on the LOCS to obtain P/F‐LOCS. A mechanical‐testing machine (Shimadzu AGS‐X, Japan) was used to test the mechanical properties of the LOCS and P/F‐LOCS. Stretching strain data at various stretching stresses were recorded (*n* = 6 samples). The scaffolds with or without cells were visualized with a scanning electron microscope (EVO LS 10, Germany) after desiccation with a supercritical point dryer and sputter‐coating with gold (*n* = 6 samples).

### 4.2 hscNPCs and hscAS isolation and culture

After legal termination of pregnancy, the aborted human fetal tissues were obtained in accordance with the Code of Ethics of the World Medical Association (Declaration of Helsinki). The use of the fetal tissue was approved by the Reproductive Study Ethics Committee of Nanjing Drum Tower Hospital, Nanjing Medical University (2018‐223‐01). All the donors gave informed consent.

The hscNPCs were obtained from human fetal spinal cord tissue according to a previously described method.^34^ The intact spinal cord tissue was carefully excised and digested with Accutase (A6964, Sigma‐Aldrich). Digestion was stopped with an appropriate volume of DMEM/F12 medium, and the cells were centrifuged, resuspended and cultured with hscNPCs proliferation medium (Table 1) in cell culture dishes.

**TABLE 1:** Composition of media

Component	Full name	Concentration	Company	Catalog number
hscNPCs proliferation media				
DMEM/F‐12	Dulbecco's modified eagle medium nutrient mixture F‐12 (Ham)		Gibco	C11330500BT
DMEM	Dulbecco's modified eagle medium		Gibco	C11965500BT
HEPES buffer	HEPES buffer	10–20 mM	Sigma‐Aldrich	83264
MEM NEAA	MEM nonessential amino acids	100×	Gibco	11140‐050
Sodium pyruvate		100×	Gibco	11360‐070
Pen strep	Penicillin streptomycin	100×	Gibco	15140‐122
Glucose	d‐(+)‐Glucose	1–2 g/100 ml	Sigma‐Aldrich	G7021
B‐27	B‐27 supplement without vitamin A	50×	Gibco	12587‐010
Insulin	Human recombinant insulin	10–49 μg/ml	Yeasen	40112ES25
Transferrin	Transferrin, apo‐, low endotoxin grade, human plasma	50–150 μg/ml	Millipore	178481
Progesterone	Progesterone	10–30 nM	Sigma‐Aldrich	P8783
Putrescine	Putrescine	50–150 μM	Sigma‐Aldrich	P7505
Sodium selenite	Sodium selenite	20–40 nM	Sigma‐Aldrich	S5261
Heparin	Heparin sodium salt from porcine intestinal mucosa	3–10 μg/ml	Sigma‐Aldrich	H3149
NT‐3	Neurotrophin‐3	10 ng/ml	PeproTech	AF‐450‐03
EGF	Epidermal growth factor	40 ng/ml	PeproTech	AF‐100‐15
bFGF	Basic fibroblast growth factor	40 ng/ml	PeproTech	AF‐450‐33
LIF	Leukemia inhibitory factor	20 ng/ml	PeproTech	AF‐300‐05
hscAS proliferation media				
DMEM/F‐12	Dulbecco's Modified Eagle Medium Nutrient Mixture F‐12 (Ham)		Gibco	C11330500BT
FBS	Fetal bovine serum	10%	Gibco	10270‐106
HBEGF	Heparin binding epidermal growth factor like growth factor	5 ng/ml	Sigma‐Aldrich,	E4643
Pen strep	Penicillin streptomycin	100X	Gibco	15140‐122
Differentiation media (hscNPC, hscAS and hscNT)				
DMEM/F‐12	Dulbecco's modified eagle medium nutrient mixture F‐12 (Ham)		Gibco	C11330500BT
DMEM	Dulbecco's modified eagle medium		Gibco	C11965500BT
HEPES buffer	HEPES buffer	10–20 mM	Sigma‐Aldrich	83264
MEM NEAA	MEM nonessential amino acids	100×	Gibco	11140‐050
Sodium pyruvate		100×	Gibco	11360‐070
Pen strep	Penicillin streptomycin	100×	Gibco	15140‐122
Glucose	d‐(+)‐Glucose	1–2 g/100 ml	Sigma‐Aldrich	G7021
B‐27	B‐27 supplement without vitamin A	50×	Gibco	12587‐010
Insulin	Human recombinant insulin	10–49 μg/ml	Yeasen	40112ES25
Transferrin	Transferrin, apo‐, low endotoxin grade, human plasma	50–150 μg/ml	Millipore	178481
Progesterone	Progesterone	10–30 nM	Sigma‐Aldrich	P8783
Putrescine	Putrescine	50–150 μM	Sigma‐Aldrich	P7505
Sodium selenite	Sodium selenite	20–40 nM	Sigma‐Aldrich	S5261
Heparin	Heparin sodium salt from porcine intestinal mucosa	3–10 μg/ml	Sigma‐Aldrich	H3149
NT‐3	Neurotrophin‐3	10 ng/ml	PeproTech	AF‐450‐03
BDNF	Brain‐derived neurotrophic factor	10 ng/ml	PeproTech	AF‐450‐02
GDNF	Glial‐derived neurotrophic factor	20 ng/ml	PeproTech	AF‐450‐10

The hscAS were harvested from electively aborted human fetuses. We carefully dissected the intact spinal cord tissue from the fetus. After removing the meninges and vascular tissues completely, the tissue was dissociated into a single‐cell suspension with nylon membranes and TrypLE (12604021, Invitrogen), and centrifuged at 500 *g* for 5 min. All cells were resuspended in hscAS proliferation culture medium (Table 1), seeded in 75 cm^2^ culture flasks, and placed into an incubator at 37°C in 5% CO_2_. When 50% confluent, the culture medium was removed, and the cells were gently rinsed three times with DMEM/F12, and fresh medium was added. The flasks were securely fixed onto an orbital shaker and shaken (200 rpm) for 6–10 h in a culture chamber. The medium and suspended cells were then removed, and adherent cells were rinsed and cultured in fresh medium. At 85% confluency, the cells were purified again, and the hscAS were passaged and used in subsequent experiments.

### 4.3 Immunostaining

Immunostaining was performed as previously described.^54^ Briefly, the samples were fixed with 4% formaldehyde for 15 min and permeabilized and blocked with 10% donkey serum containing 0.03% Triton X‐100 for 1 h at room temperature (RT). The cells were stained with primary antibodies overnight at 4°C and then incubated with the corresponding secondary antibody for 1 h at RT. Finally, images were captured using a confocal microscope (Leica Microsystems, Germany). The antibodies used in this study are listed in Table 2.

**TABLE 2:** Antibodies

Name	Company	Catalog number	Host	Dilution ratio
Nestin	NEUROMICS	MO15012	Mouse	1:200
SOX2	GeneTex	GTX101507	Rabbit	1:500
PAX3	Abcam	ab180754	Rabbit	1:200
Ki67	Abcam	ab15580	Rabbit	1:500
Tuj‐1	GeneTex	GTX631836	Mouse	1:500
Tuj‐1	Abcam	ab18207	Rabbit	1:500
DCX	Abcam	ab113435	Goat	
MAP2	Sigma‐Aldrich	M4403	Mouse	1:250
MAP2	Abcam	ab32454	Rabbit	1:250
NF70	Sigma‐Aldrich	N4142	Rabbit	1:200
NeuN	Abcam	ab177487	Rabbit	1:500
GFAP	Abcam	ab7260	Rabbit	1:500
GFAP	Abcam	ab134436	chicken	1500
OLIG2	Millipore	AB9610	Rabbit	1:500
S100β	Abcam	ab52642	Rabbit	1:500
GLAST	Abcam	ab416	Rabbit	1:500
S100A4	Biolegened	810,101	Rabbit	1:200
SOX‐9	R&D Systems	AF3075	Goat	1:200
P2Y12	GeneTex	GTX54796	Rabbit	1:1000
Iba1	Abcam	ab15690	Mouse	1:500
GFP	Abcam	ab290	Rabbit	1:500
GFP	GeneTex	GTX13970	Chicken	1:500
vGLUT1	Millipore	AB5905	Guinea Pig	1:300
GABA	MilliporeSigma	A0310	Mouse	1:250
ChAT	Abcam	ab18736	Sheep	1:250
PSD95	Abcam	ab18258	Rabbit	1:200
SYN	Millipore	MAB329	Mouse	1:200
5‐HT	ImmunoStar	20,080	Rabbit	1:200
CS56	Abcam	ab11570	Mouse	1:500
CD68	Abcam	ab125212	Rabbi	1:500
RECA	Abcam	ab22492	Mouse	1:500

### 4.4 Cell viability and size analyses

The hscNPCs and hscAS were stained with acridine orange/propidium iodide (PI), and cell viability and size analyses were performed using a Cellometer AUTO2000 (Nexcelom, Germany). Data were analyzed with Graphpad Prism 8.0.2 (263) (*n* = 6 samples).

### 4.5 Growth curve and population doubling time

A CCK‐8 kit (HY‐K0301, MedChemExpress) was used for cell counting of hscNPCs and hscAS. The absorbance value of each well was measured with a spectrophotometer at 450 nm every day for 9 days. A growth curve was drawn with Graphpad Prism 8.0.2 (263). Population doubling time (PDT) was calculated with the following formula: PDT = *t* lg2/lg(*N*_t_/*N*_o_), where *t* is the culture time, *N*_t_ is the final cell number and *N*_o_ is the initial cell number (*n* = 3 samples).

### 4.6 Karyology

hscNPCs and hscAS at passage 5 were treated with 2 μg/ml colcemid solution (10092013, Invitrogen) for 4 h at 37°C following metaphase arrest. Then cells were dissociated and resuspended in a hypotonic solution of 0.00375 M KCl for 10 min at RT. Centrifugation and fixation were repeat 3 times. Dried samples were stained with DAPI mounting medium (ZLI‐9557, ZSGB‐BIO) and harvest of chromosome clusters was confirmed under a fluorescence microscope. The definition of clones and the description of karyotypes were in accordance with International System for Human Cytogenetic Nomenclature (ISCN, 2006) (*n* = 3 samples).

### 4.7 Tumorigenicity test

Animal experiments and all associated procedures were performed following the Guide for the Care and Use of Laboratory Animals of the National Institutes of Health, and were approved by the Institute of Genetics and Developmental Biology at the Chinese Academy of Sciences (AP2018014).

A total of 30 nude mice (female, 4 weeks old) were obtained from Vitalriver (China) and randomly divided into three groups (10 mice/group): positive (Hela) control group, hscNPCs group and hscAS group. Random numbers were generated using the standard = RANDBETWEEN(1, 30) function in Microsoft Excel. All mice housed in temperature and humidity controlled animal quarters with a 12 h light/dark cycle. Each mouse was injected subcutaneously with a total of 1 × 10^7^ cells. All animals were observed for 4 months.

### 4.8 Cell cycle

Nuclear DNA content was measured using a nuclear detection kit (KGA511, Keygentec) and flow cytometric analysis. Briefly, hscNPCs and hscAS were dissociated and collected, washed with PBS, and fixed in cold 70% ethanol overnight at 4°C. The cells were thereafter washed once with PBS, resuspended in staining buffer (PI/RNase A) for 20 min at RT, and analyzed using Attune NxT Focusing Cytomete AFC2 (Life Technologies, USA) (*n* = 3 samples).

### 4.9 Transfection of hscNPCs and hscAS with lentivirus

The hscNPCs were transfected with lentivirus carrying green fluorescent protein gene (GFP; Miga Technology Co. Ltd., China) to obtain GFP‐hscNPCs (Figure S1a,b). The hscAS were transfected with lentivirus carrying a red fluorescent protein gene (RFP; Miga Technology Co. Ltd., China) to obtain RFP‐hscAS (Figure S4a,b). Briefly, at 40–50% confluence, the supernatant was replaced with 7 ml of fresh culture medium containing lentivirus. Then, 10 h later, 8 ml of culture medium was added and incubated for another 72 h before passaging.

### 4.10 hscNT construction

The hscNPCs or hscAS were dissociated and seeded on the P/F‐LOCS, and the appropriate hscNPCs proliferation medium was added until the material was covered. The material and cells were incubated for 2 h, and then, another 2 ml of proliferation medium was added. For hscNT construction, hscNPCs was firstly seeded on the P/F‐LOCS, and then, the hscAS were seeded, as described above. Finally, cell proliferation medium was added for further coculture. After 3 days, the hscNPCs, hscAS or hscNT were transferred into the differentiation medium (Table 1) for in vitro differentiation analysis, or they were transplanted to repair SCI in rats. The experimental groups were hscNPCs alone (2 × 10^6^), hscAS alone (2 × 10^6^) and hscNT (1.6 × 10^6^ hscNPCs +4 × 10^5^ hscAS).

### 4.11 Cell viability

For viability assays, hscAS on the P/F‐LOCS were treated with 2 μM calcein AM and 4 μM ethidium homodimer‐1 (R37601, Thermo Fisher Scientific) in differentiation medium for 15 min, and then observed under a Leica TCS SP8 confocal microscope (Leica Microsystems). Cell viability was calculated using ImageJ software (*n* = 3 samples).

### 4.12 Cell growth

Images of the hscNT were captured under a fluorescence microscope (Axiovert200, ZEISS,) at 7, 15, and 30 days. The outgrowth length of hscNPCs was measured using ImageJ software (*n* = 6 samples).

### 4.13 Calcium imaging

Live‐cell calcium imaging was performed using an Andor Dragonfly 200 confocal laser scanning system, equipped with a temperature and CO_2_ control module. For calcium imaging, Fluo‐4 AM (F14201, Life Technologies) was prepared according to the manufacturer's instructions and was applied to the sample for 60 min in darkness. The supernatant was then removed, and the sample was washed with HBSS three times. Imaging was performed at 494 nm excitation. Photos were taken every 2 s for 20 min (*n* = 3 samples).

### 4.14 Surgery procedures and transplantation

Animal experiments and all associated procedures were performed following the Guide for the Care and Use of Laboratory Animals of the National Institutes of Health, and were approved by the Institute of Genetics and Developmental Biology at the Chinese Academy of Sciences (AP2018014).

Female Sprague–Dawley rats (50, 200–220 g) were obtained from Vitalriver (China) and randomly divided into five groups (10 rats/group): SCI (no transplantation), SCI + L (transplantation of the P/F‐LOCS only), SCI + L + A (P/F‐LOCS loaded with hscAS), SCI + L + P (P/F‐LOCS loaded with hscNPCs) and SCL + L + A + P (4:1 ratio of hscNPCs and hscAS cocultured on the P/F‐LOCS [hscNT]). Random numbers were generated using the standard = RANDBETWEEN (1, 50) function in Microsoft Excel. All rats housed in temperature and humidity controlled animal quarters with a 12 h light/dark cycle.

The surgery was performed according to our previous report^54^ with slight modification. Following anesthetization, a 3‐mm segment of the spinal cord was completely removed after laminectomy. An empty scaffold or a scaffold loaded with a total of 1 × 10^6^cells was transplanted after stopping the bleeding (Figure 3a). Then, the musculature and skin were closed in separate layers with sutures. After surgery, the bladders were manually emptied twice daily until the urination reflex recovered. All rats were given intraperitoneal injections of cyclosporine A (10 mg/kg, daily), starting at 7 days prior to surgery, until sacrifice.

### 4.15 Assessment of locomotor performance

The BBB scores were assessed weekly during 2 months by two independent observers blinded to group identity (*n* = 6 rats). Eight weeks after transplantation, CMEPs were recorded as described in our previous study^34^ (*n* = 4 rats).

### 4.16 Perfusion and immunohistochemistry

After deep anesthetization, rats were intracardially perfused with physiological saline and 4% paraformaldehyde. Then, the spinal cord was dissected and fixed overnight before dehydrating in 20% sucrose, followed by 30% sucrose. Longitudinal sections of the spinal cord were cut at 18‐μm thickness and stored at −80°C for immunofluorescence staining.

After rinsing, permeabilization and blocking, sections were incubated with primary antibodies at 4°C overnight, and then with the appropriate secondary antibody for 1 h at RT (Table 2). DAPI was used for nuclear staining when necessary. Slides were examined under a confocal microscope. The antibodies used in this study are listed in Table 2 (*n* = 3 rats).

### 4.17 Statistical analyses

Data are presented as mean ± SEM. Graphpad Prism 8.0.2 (263) was used for all statistical analyses. When more than two sets of data were compared, one‐way analysis of variance was performed, and Tukey's test was used for post hoc analysis to correct for multiple comparisons. Two‐group comparisons were analyzed using Student's *t*‐test. *p*‐values <0.05 were considered significant.

## 5 CONCLUSIONS

In summary, we fabricated a novel centimeter‐scale hscNT with multiple cell types and a linearly ordered structure. The efficiency of the hscNT for SCI repair was investigated. The hscNT created a favorable microenvironment for endogenous neural regeneration, and it structurally integrated with host neural elements, thereby promoting motor functional recovery. Engineered human spinal cord implants containing astrocytes and neurons assembled on axon guidance scaffolds may therefore have therapeutic potential for SCI.

## AUTHOR CONTRIBUTIONS

**Chen Jin:** Conceptualization (equal); data curation (equal); investigation (equal); methodology (lead); software (lead); validation (lead); visualization (lead); writing – original draft (lead); writing – review and editing (lead). **Yayu WU:** Data curation (equal); methodology (equal); visualization (equal). **Haipeng Zhang:** Methodology (equal); software (equal); visualization (equal). **Bai Xu:** Data curation (equal); formal analysis (equal); methodology (equal). **Wenbin Liu:** Methodology (supporting); resources (equal). **Chunnan Ji:** Methodology (supporting); resources (equal). **Panpan Li:** Methodology (supporting); resources (equal). **Zhenni Chen:** Methodology (supporting); software (equal). **Bing Chen:** Investigation (equal); supervision (equal); validation (equal). **Jiayin Li:** Funding acquisition (equal); supervision (equal). **Xianming Wu:** Funding acquisition (supporting); visualization (equal). **Yali HU:** Conceptualization (supporting); resources (equal); supervision (supporting). **Peipei Jiang:** Resources (equal); supervision (equal). **Zhifeng Xiao:** Conceptualization (supporting); investigation (equal); methodology (equal); supervision (equal); visualization (equal). **Yannan Zhao:** Conceptualization (equal); funding acquisition (equal); methodology (equal); supervision (equal); writing – original draft (equal); writing – review and editing (equal). **Jianwu Dai:** Conceptualization (lead); funding acquisition (lead); supervision (lead).

## CONFLICT OF INTEREST

The authors declare that they have no competing interests.

## Supporting information


**Figure S1.** Loading of hscNPCs onto the P/F‐LOCS. (a) hscNPCs transfected with lentivirus carrying green fluorescent protein (GFP). (b) The lentivirus infected hscNPCs with an efficiency of up to 85 ± 3.12% (*n* = 6 images). (c) Images of hscNPCs in the LOCS, LOCS modified with 10% poly‐l‐ornithine (P‐LOCS), LOCS modified with 1% fibronectin (F‐LOCS) and LOCS modified with 10% poly‐l‐ornithine and 1% fibronectin (P/F‐LOCS). (d) Quantification of GFP^+^ signal intensity per visual field (*n* = 5 images). Error bars represent standard error. (e) Representative image of hscNPCs residing on the P/F‐LOCS. One‐way analysis of variance, with Tukey's test for post hoc analysis to correct for multiple comparisons. ****p < 0.0001.

**Figure S2.** Characterization of hscNPCs. (a) hscNPCs exhibited similar viability at passages 0, 1, 2, 3, 4, and 5 (*n* = 6 samples). (b) Representative growth curve of cultured hscNPCs at passages 1, 3, and 5. (c) The population doubling time of hscNPCs increased slightly with passages, but there was no significant difference (*n* = 3 samples). (d) Cell cycle showing hscNPC proliferation. (e) Similar cell size of hscNPCs at passages 0, 1, 2, 3, 4 and 5 (*n* = 6 samples). (f) The tumorigenicity study of hscNPCs compared with HeLa cells. (g) Normal karyology of hscNPCs at passage 5. (h) hscNPCs rarely expressed MAP2, GFAP or OLIG2. (i) Bar graph showing percentages of cells positive for the different markers (*n* = 9 images). Error bars represent standard error, and multigroup comparisons were analyzed using one‐way analysis of variance with Tukey's test. P, passage; d, day.

**Figure S3.** Characterization of hscAS. (a) Purification of primary hscAS. (b) Microglial (Iba1^+^) cells were present among primary hscAS before purification. (c) There are no neurons (DCX^+^ and MAP2^+^), oligodendrocyte precursor cells (Olig2^+^), fibrocytes (S100A4^+^) or microglia (Iba1^+^) among hscAS after purification. (d,e) hscAS exhibited similar cell viability (d) and cell size (e) at passages 0, 1, 2, 3, and 4 (*n* = 6 samples). (F) Representative growth curve of hscAS at passages 1, 3, and 5. (g) Population doubling time of hscAS increased slightly with increasing passage number, but there was no significant difference (*n* = 3 samples). (h) Cell cycle showing hscAS proliferation. (i) Normal karyology of hscAS at passage 5. (j) The tumorigenicity study of hscAS compared with HeLa cells. Error bars represent standard error, and multigroup comparisons were analyzed using one‐way analysis of variance with Tukey's test. P, passage; d, day.

**Figure S4.** Diagram of the structure of the hscNT. (a) hscAS transfected with lentivirus carrying red fluorescent protein (RFP). (b) The lentivirus infected hscAS with an efficiency of up to 90.90 ± 0.73% (*n* = 6 images). (c) Immunofluorescence image showing that both GFP‐hscNPCs and RFP‐hscAS grew in an orderly fashion along the P/F‐LOCS.

**Figure S5.** hscAS promote hscNPCs adhesion, survival and neurite growth. (a) Images of GFP‐hscNPCs cultured on the P/F‐LOCS alone or with hscAS at 7, 15, and 30 days. (b) The percentages of GFP^+^ signal intensity/material area per visual field (*n* = 6 images), and the quantification of mean neurite length of GFP‐hscNPCs (*n* = 30 cells). (c) hscAS promoted hscNPCs adhesion on a dish. (d) hscAS promoted neurite growth from GFP‐hscNPCs on the dish. Error bars represent standard error. One‐way analysis of variance, with Tukey's test for post hoc analysis to correct for multiple comparisons. **p < 0.01; ***p < 0.001; ****p < 0.0001.

**Movie S1:** Cells in the hscNT showed spontaneous calcium surges during Fluo‐4 calcium live cell imaging.

**Movie S2:** Cells in the hscNPCs cultured alone did not show spontaneous calcium surges during Fluo‐4 calcium live cell imaging.

## Data Availability

The data that support the findings of this study are available from the corresponding author upon reasonable request.
