# Supplementary figures and images for: Spinal cord tissue engineering using human primary neural progenitor cells and astrocytes
Source: Bioeng Transl Med. 2022 Nov 9;8(2):e10448. doi: 10.1002/btm2.10448 (PMC10013752; doi:10.1002/btm2.10448)

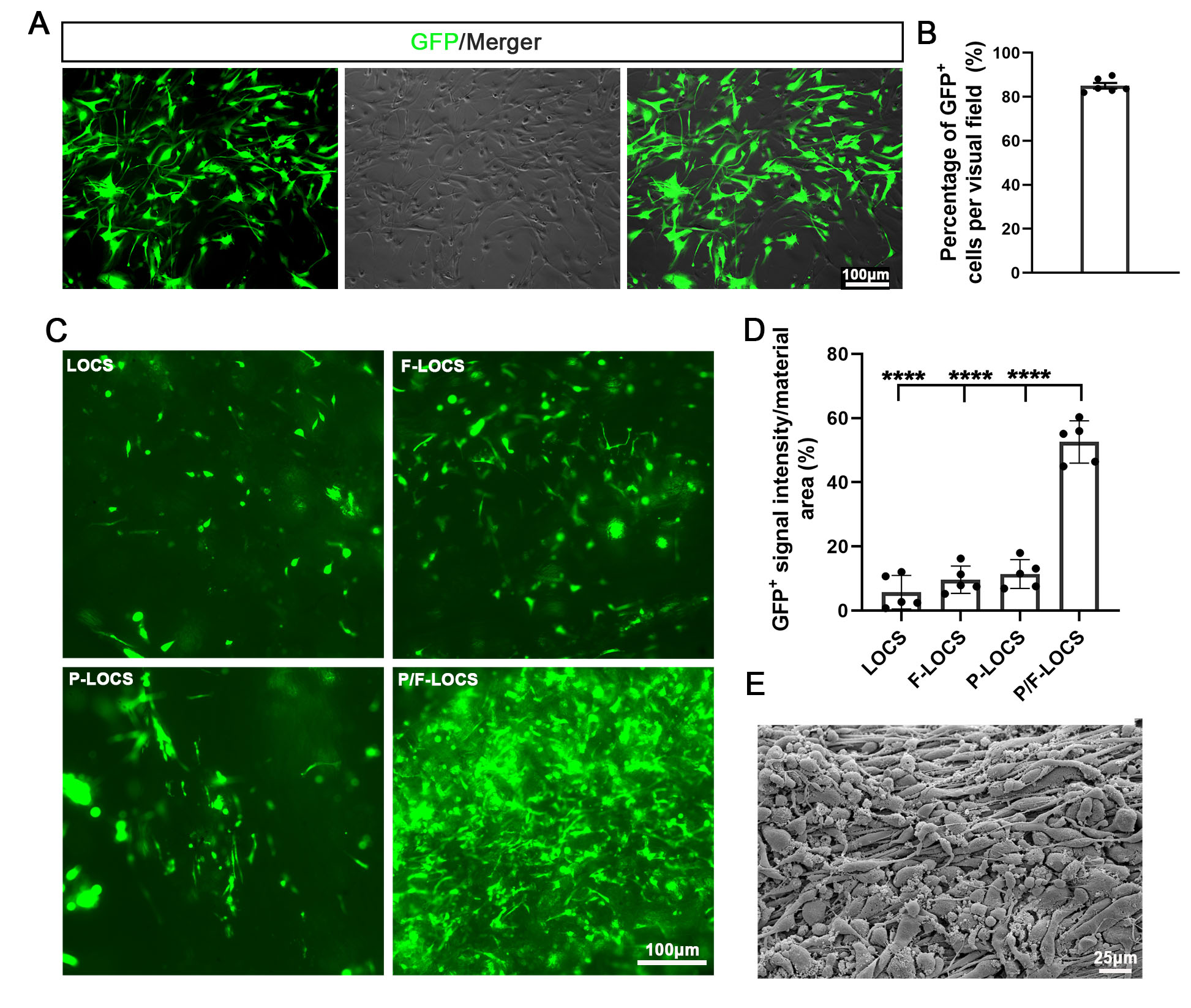

Supplement: Supplementary file 1 — Figure S1. Loading of hscNPCs onto the P/F‐LOCS. (a) hscNPCs transfected with lentivirus carrying green fluorescent protein (GFP). (b) The lentivirus infected hscNPCs with an efficiency of up to 85 ± 3.12% (n = 6 images). (c) Images of hscNPCs in the LOCS, LOCS modified with 10% poly‐l‐ornithine (P‐LOCS), LOCS modified with 1% fibronectin (F‐LOCS) and LOCS modified with 10% poly‐l‐ornithine and 1% fibronectin (P/F‐LOCS). (d) Quantification of GFP+ signal intensity per visual field (n = 5 images). Error bars represent standard error. (e) Representative image of hscNPCs residing on the P/F‐LOCS. One‐way analysis of variance, with Tukey's test for post hoc analysis to correct for multiple comparisons. ****p < 0.0001. [file BTM2-8-e10448-s004.jpg]

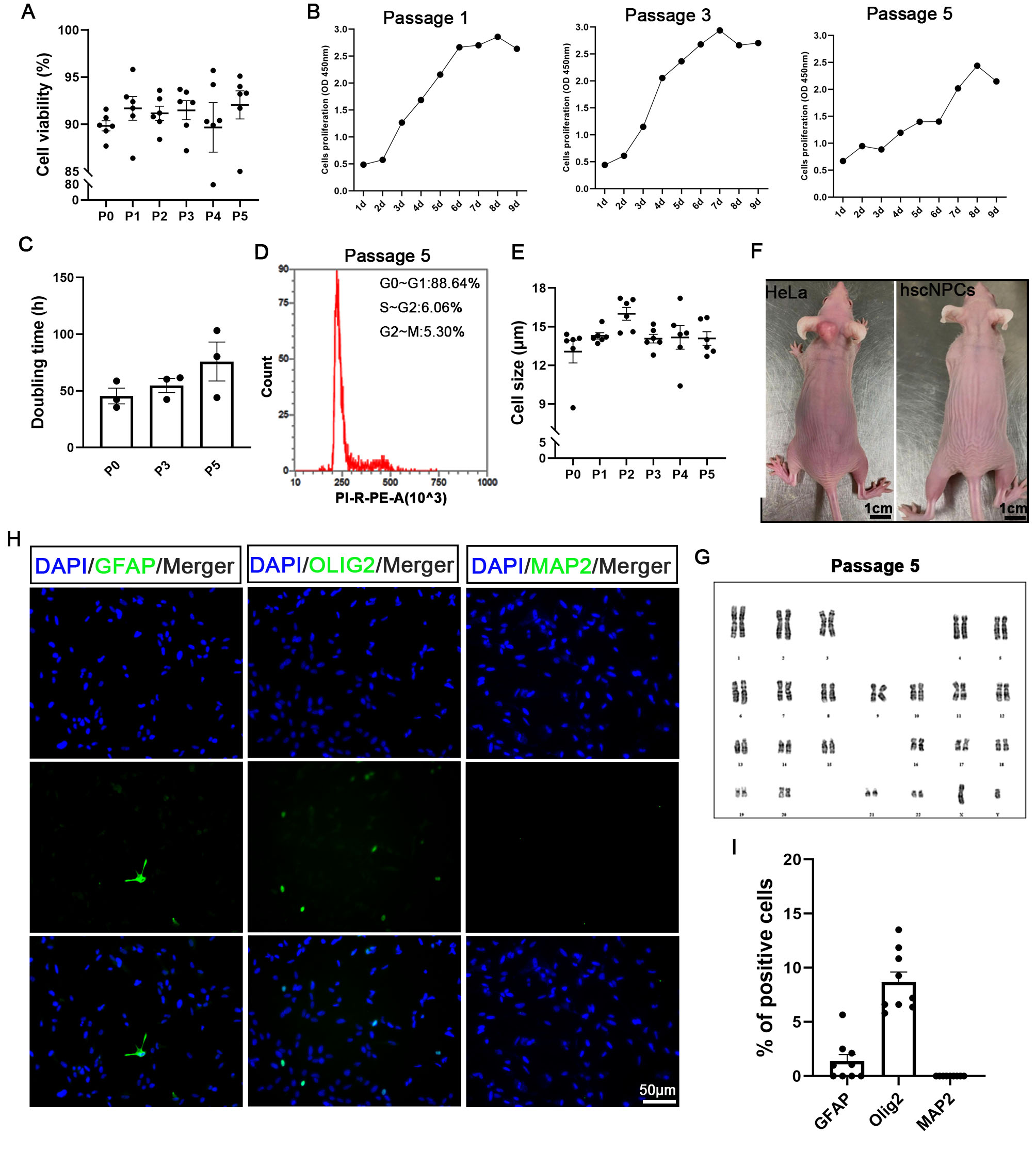

Supplement: Supplementary file 2 — Figure S2. Characterization of hscNPCs. (a) hscNPCs exhibited similar viability at passages 0, 1, 2, 3, 4, and 5 (n = 6 samples). (b) Representative growth curve of cultured hscNPCs at passages 1, 3, and 5. (c) The population doubling time of hscNPCs increased slightly with passages, but there was no significant difference (n = 3 samples). (d) Cell cycle showing hscNPC proliferation. (e) Similar cell size of hscNPCs at passages 0, 1, 2, 3, 4 and 5 (n = 6 samples). (f) The tumorigenicity study of hscNPCs compared with HeLa cells. (g) Normal karyology of hscNPCs at passage 5. (h) hscNPCs rarely expressed MAP2, GFAP or OLIG2. (i) Bar graph showing percentages of cells positive for the different markers (n = 9 images). Error bars represent standard error, and multigroup comparisons were analyzed using one‐way analysis of variance with Tukey's test. P, passage; d, day. [file BTM2-8-e10448-s001.jpg]

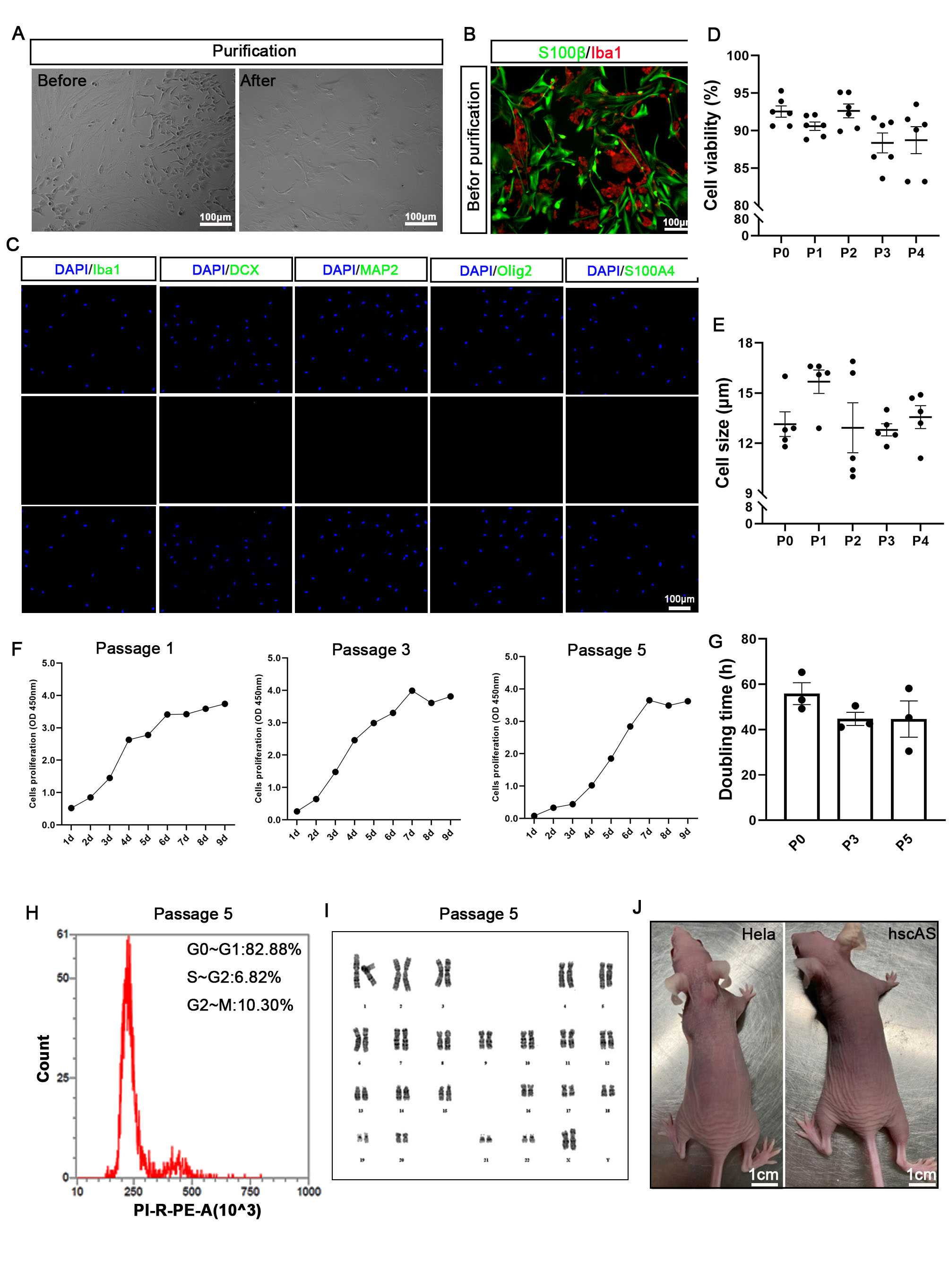

Supplement: Supplementary file 3 — Figure S3. Characterization of hscAS. (a) Purification of primary hscAS. (b) Microglial (Iba1+) cells were present among primary hscAS before purification. (c) There are no neurons (DCX+ and MAP2+), oligodendrocyte precursor cells (Olig2+), fibrocytes (S100A4+) or microglia (Iba1+) among hscAS after purification. (d,e) hscAS exhibited similar cell viability (d) and cell size (e) at passages 0, 1, 2, 3, and 4 (n = 6 samples). (F) Representative growth curve of hscAS at passages 1, 3, and 5. (g) Population doubling time of hscAS increased slightly with increasing passage number, but there was no significant difference (n = 3 samples). (h) Cell cycle showing hscAS proliferation. (i) Normal karyology of hscAS at passage 5. (j) The tumorigenicity study of hscAS compared with HeLa cells. Error bars represent standard error, and multigroup comparisons were analyzed using one‐way analysis of variance with Tukey's test. P, passage; d, day. [file BTM2-8-e10448-s007.jpg]

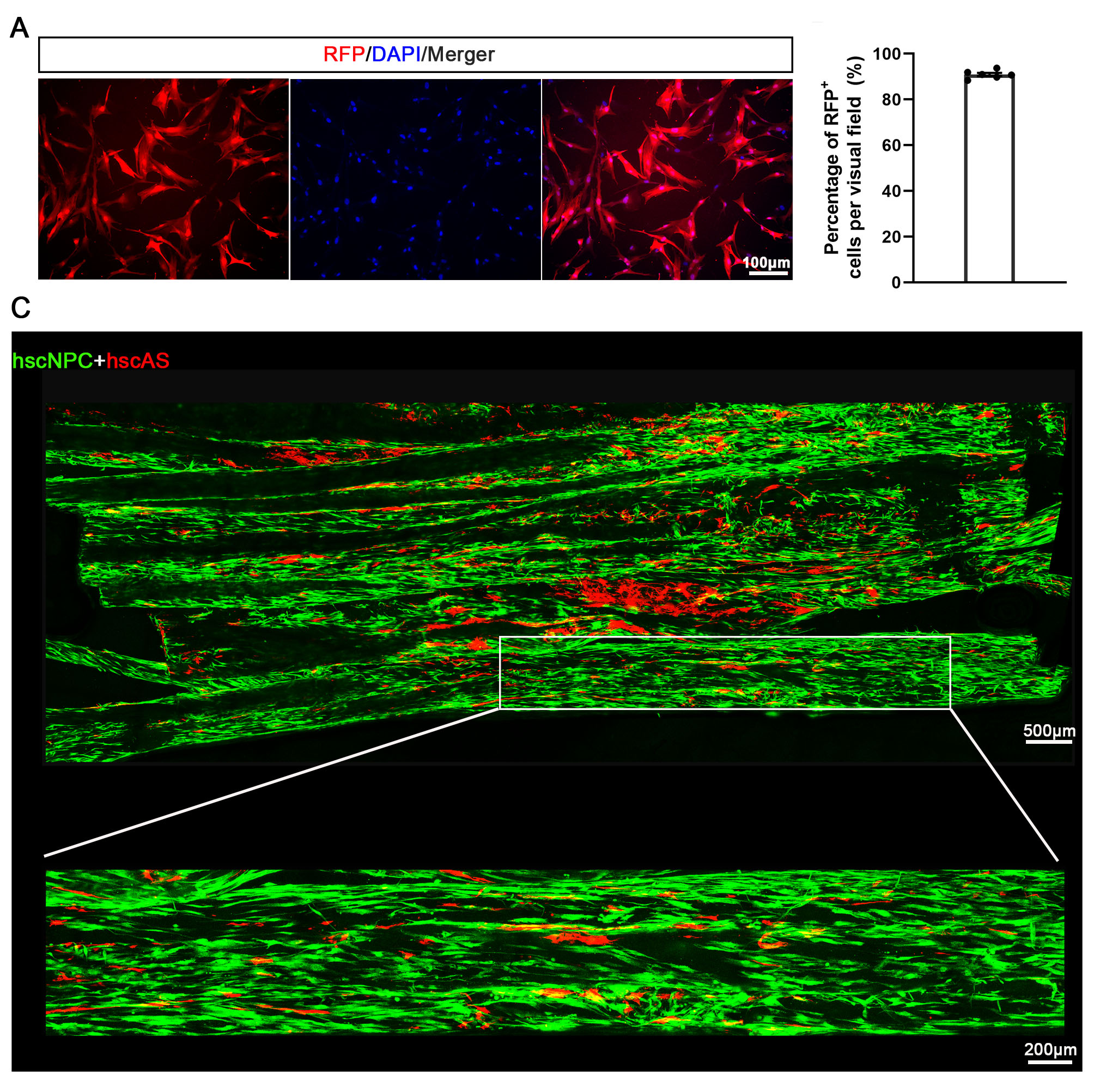

Supplement: Supplementary file 4 — Figure S4. Diagram of the structure of the hscNT. (a) hscAS transfected with lentivirus carrying red fluorescent protein (RFP). (b) The lentivirus infected hscAS with an efficiency of up to 90.90 ± 0.73% (n = 6 images). (c) Immunofluorescence image showing that both GFP‐hscNPCs and RFP‐hscAS grew in an orderly fashion along the P/F‐LOCS. [file BTM2-8-e10448-s006.jpg]

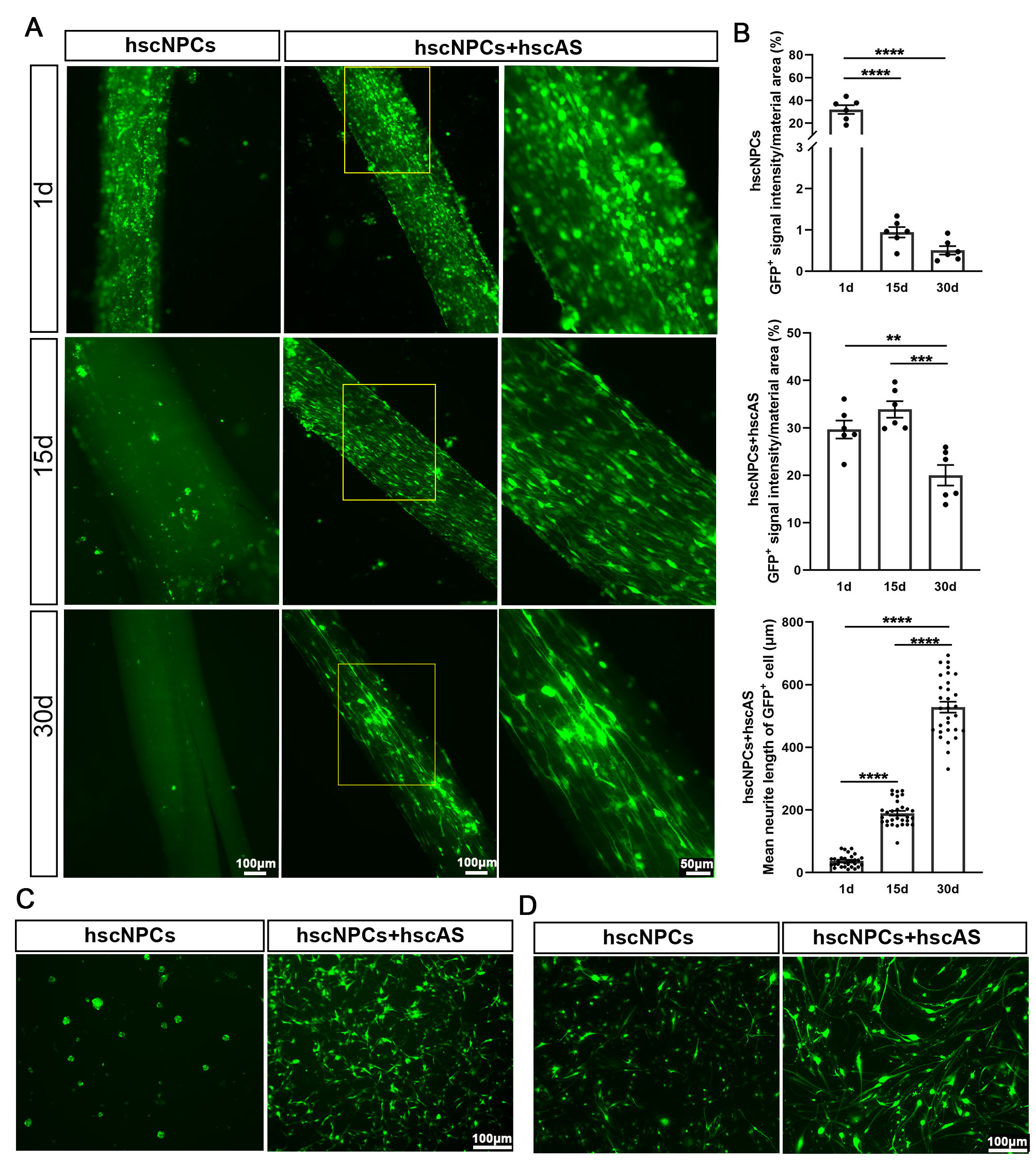

Supplement: Supplementary file 5 — Figure S5. hscAS promote hscNPCs adhesion, survival and neurite growth. (a) Images of GFP‐hscNPCs cultured on the P/F‐LOCS alone or with hscAS at 7, 15, and 30 days. (b) The percentages of GFP+ signal intensity/material area per visual field (n = 6 images), and the quantification of mean neurite length of GFP‐hscNPCs (n = 30 cells). (c) hscAS promoted hscNPCs adhesion on a dish. (d) hscAS promoted neurite growth from GFP‐hscNPCs on the dish. Error bars represent standard error. One‐way analysis of variance, with Tukey's test for post hoc analysis to correct for multiple comparisons. **p < 0.01; ***p < 0.001; ****p < 0.0001. [file BTM2-8-e10448-s003.jpg]
